# Diagnostic value of urine sTREM-1 for sepsis and relevant acute kidney injuries: a prospective study

**DOI:** 10.1186/cc10508

**Published:** 2011-10-24

**Authors:** Long-xiang Su, Lin Feng, Jie Zhang, Yong-jiu Xiao, Yan-hong Jia, Peng Yan, Dan Feng, Li-xin Xie

**Affiliations:** 1 Department of Respiratory Diseases, Chinese PLA General Hospital, 28 Fuxing Rd, Beijing, 100853, China; 2 Department of Medical Statistics, Chinese PLA General Hospital, 28 Fuxing Rd, Beijing, 100853, China; 3 Medical School, Nankai University, 94 Weijin Rd, Tianjin, 300071, China

**Keywords:** urine, soluble triggering receptor expressed on myeloid cells-1(sTREM-1), sepsis, severity, prognosis, acute kidney injury (AKI), sensitivity, specificity

## Abstract

**Introduction:**

We explored the diagnostic value of a urine soluble triggering receptor expressed on myeloid cells-1 (sTREM-1) for early sepsis identification, severity and prognosis assessment, and for secondary acute kidney injury (AKI). We compared this with white blood cell (WBC) counts, serum C-reactive protein (CRP), serum procalcitonin (PCT), urine output, creatinine clearance (CCr), serum creatinine (SCr), and blood urea nitrogen (BUN).

**Methods:**

We enrolled 104 subjects admitted to the ICU: 16 cases with systemic inflammatory response syndrome (SIRS); 35 with sepsis and 53 with severe sepsis. Results for urine sTREM-1, WBC, serum CRP and serum PCT were recorded on days 1, 3, 5, 7, 10, and 14. For 17 sepsis cases diagnosed with secondary AKI, comparisons between their urine sTREM-1, urine output, CCr, SCr and BUN at diagnosis and 48 h before diagnosis were made.

**Results:**

On the day of admission to the ICU, and compared with the SIRS group, the sepsis group exhibited higher levels of urine sTREM-1 and Acute Physiologic Assessment and Chronic Health Evaluation II (APACHE II) scores (*P *< 0.05). Areas under the curve (AUC) shaped by the scores were 0.797 (95% CI 0.711 to 0.884) and 0.722 (95% CI 0.586 to 0.858), respectively. On days 1, 3, 5, 7, 10, and 14, urine sTREM-1, serum PCT and WBC levels registered higher in the severe sepsis group in contrast to the sepsis group (*P *< 0.05). Urine sTREM-1 and serum PCT levels continuously increased among non-survivors, while WBC and serum CRP levels in both groups declined. For 17 patients with AKI, urine sTREM-1, SCr and BUN levels at 48 h before AKI diagnosis were higher, and CCr level was lower than those for non-AKI subjects (*P *< 0.05). AUC for urine sTREM-1 was 0.922 (95% CI 0.850 to 0.995), the sensitivity was 0.941, and the specificity was 0.76 (based on a cut-off point of 69.04 pg/ml). Logistic regression analysis showed that urine sTREM-1 and severity were risk factors related to AKI occurrence.

**Conclusions:**

Besides being non-invasive, urine sTREM-1 testing is more sensitive than testing WBC, serum CRP, and serum PCT for the early diagnosis of sepsis, as well as for dynamic assessments of severity and prognosis. It can also provide an early warning of possible secondary AKI in sepsis patients.

**Trial Registration:**

ClinicalTrial.gov identifier NCT01333657

## Introduction

Sepsis is a major factor contributing to ICU admissions and patient deaths. A recent epidemiologic study in North America found that the incidence of sepsis was 3% in the total population and that the mortality rate for septic shock patients was as high as 50% [1]. Because of its rapid progression, the disease might, within a relatively short period of time, lead to secondary multiple organ dysfunction syndrome (MODS) and endanger a patient's life [2]. Acute kidney injury (AKI) is well known to be associated with longer length of stay, morbidity and mortality in adults [3,4]. Sepsis-related kidney failure occurs in about 19% of sepsis patients. This incidence may reach 23% among severe sepsis patients and is 51% among those with positive blood cultures, with a mortality rate of 70%, much higher than among patients who are free from these complications [5]. Clinical practice has shown that for patients with severe sepsis and septic shock, an early, effective intervention can clearly improve prognosis and reduce mortality [6]. For this reason, it is clinically important to identify indicators that can be used for the early diagnosis and prognosis of sepsis and its related AKI.

The triggering receptor expressed on myeloid cells-1(TREM-1) is a recently discovered member of the immunoglobulin superfamily of receptors that is expressed on polymorphonuclear granulocytes and mature monocytes. Bacterial or fungal infections may induce its expression. sTREM-1 is a soluble form of TREM-1 that may be released into body fluids upon the up-regulated expression of TREM-1 [7]. An increasing number of studies indicate that there are increased levels of sTREM-1 in body fluid samples for the following diseases and conditions: sepsis, pneumonia, pleural effusion, septic arthritis, meningitis, peritonitis and uterine cavity infection [8-14]. This suggests that sTREM-1 may be a valuable diagnostic indicator for making distinctions between infectious and non-infectious diseases. It has also been found that infectious shock patients have high levels of serum sTREM-1 that are closely related to the severity of infection [15,16]. With regard to sepsis prognosis, dynamic changes in serum sTREM-1 may provide warnings concerning survival or fatality [17,18].

Inflammation is now believed to play a major role in the pathophysiology of AKI [19,20]. It is hypothesized that the initial insult results in morphological and/or functional changes in vascular endothelial cells and/or in tubular epithelium in sepsis models [21,22]. Then, leukocytes including neutrophils, macrophages, natural killer cells, and lymphocytes infiltrate into the injured kidneys and induce the generation of inflammatory mediators [23,24]. Whether urine sTREM-1 could be detected and its significance in sepsis and AKI have not been reported yet.

The present study focused on the value of urine sTREM-1 for sepsis identification, severity and prognosis assessments, and potential sepsis-related AKI. Comparisons are also made among sTREM-1, WBC counts, serum CRP, serum procalcitonin (PCT), urine output, creatinine clearance (CCr), SCr, and blood urea nitrogen (BUN) among sepsis patients, in an effort to define relevant diagnostic values.

## Materials and methods

### Study subjects

All subjects were selected from among inpatients who were hospitalized between March 2010 and March 2011 in the Respiratory ICU, Surgical ICU and Emergency ICU of Chinese People's Liberation Army (CPLA) General Hospital. Based on the 1991 ACCP/SCCM Sepsis Directory [25] and the diagnosis criteria advanced by the 2001 International Sepsis Definition Conference [26], the subjects were divided into a systemic inflammatory response syndrome (SIRS) group and a sepsis group, with the occurrence of infection as the norm for division. Based on the severity of their conditions, the latter was sub-divided into a sepsis group and a severe sepsis group (severe sepsis and septic shock). Sepsis patients were also divided into a survivor group and a non-survivor group, with a 28-day survival as the dividing line. This study focuses on dynamic changes of different indicators. For this, all the patients involved stayed in ICUs ≥ 14 days or died within 14 days.

The 2006 Acute Kidney Injury Network (AKIN) defines AKI as different degrees of abnormal kidney structure and function as well as abnormal kidney damage signs lasting not more than three months, and manifested clinically by blood, urine and tissue tests and imaging studies [27], characterized by a 48 h absolute increase in serum creatinine (SCr) ≥ 26.4 μmol/L (0.3 mg/dL) or a percentage increase ≥ 50%, or a ≥ 6 h decline in urine output to ≤ 0.5 ml/kg·h.

Patients excluded: (1) were younger than 18 years of age; (2) suffered from anuria; (3) contracted acquired immunodeficiency syndrome; (4) had reduced polymorphonuclear granulocyte counts (< 500 μL^-1^); (5) were receiving dialysis treatment for chronic kidney disease; (6) died within 24 h after admission into the ICU, or refused to participate in the study, or declined treatment during the period of observation. Patients or their family members were fully informed of the study details and signed informed consent forms of their own accord. This study was approved by the Ethics Committee of the CPLA General Hospital (project No.20090923-001) and was registered with the U.S. National Institutes of Health Clinical Trials Register (NCT 01333657).

### Data collection

Upon admission into the ICU, the following items were recorded for each patient: age, gender, chief complaints for admission, symptoms, APACHE II scores, Sequential Organ Failure Assessment (SOFA) scores, white blood cell (WBC) counts, serum c-reactive protein (CRP), PCT, urine sTREM-1, CCr, SCr, BUN, urine output, mechanical ventilation, continuous renal replacement treatment (CRRT), AKI, etiological factors and underlying diseases. A record was also kept of 28-day survivals.

### Assays

Within 24 h (the first day of study) after ICU admission, blood and urine samples were collected. Tests on intravenous blood and urine samples were repeated on the mornings of days 3, 5, 7, 10, and 14. Blood was centrifuged at 3,000 rpm for 15 minutes, and urine at 2,000 rpm for 5 minutes. The supernatants were transferred to Eppendorf tubes and stored at -80°C. All the specimens were re-numbered before the experiment. We saw to it that each step was blind to the staffers involved. sTREM-1 was examined by a double antibody sandwich ELISA (Quantikine Human TREM-1 Immunoassay ELISA Kit, R & D Systems, Minneapolis, MN, USA, product number DTRM10B); CRP, by scattering turbidimetry (Cardio Phase hsCRP, Siemens, Munich, Germany); PCT, by enzyme-linked fluorescence analysis (ELFA, VIDAS BRAHMS PCT kit, BioMérieux, Marcy-l'Étoile, France) and SCR and BUN, by an enzymatic method (CREA plus & UREA/BUN reagent, Roche Modular, Mannheim, Germany). ELISA was performed in duplicate and other assays were done in strict accordance with the manufacturers' instructions.

### Statistical analysis

Results for continuous variables with normal distributions, including age, WBC counts, urine output, CCr, APACHE II scores and SOFA scores are given as means ± standard deviations (SD). Student's *t*-test was used to compare means between two groups. Analysis of variance (ANOVA) was used to compare means among multiple groups. Results for continuous variables that were not normally distributed, including sTREM-1, CRP, PCT, SCr and BUN, are given as medians (25^th ^and 75^th ^percentiles) and were compared using non-parametric tests. Results for qualitative variables were expressed as percentages and compared between groups using a Chi-square test. Areas under receiver operating characteristic curves were used to evaluate how well the model distinguished sepsis patients from SIRS patients, and AKI patients from non-AKI patients. Multivariate analysis used a logistic regression model to estimate the odds ratio for AKI and the 95% confidence interval (CI). Stepwise and forward selection procedures were used to iteratively select variables possibly related to AKI. To be entered into this model, a *P *< 0.05 from univariate analysis was required. Risk factors related to disease were explored using multivariate logistic regression. Statistical analyses were conducted by SPSS 16.0 (SPSS, Chicago, IL, USA) and a two-tailed *P *< 0.05 was considered significant.

## Results

### Subject characteristics

A total of 205 patients with SIRS were involved in the study. Of these, 21 patients were later excluded according to the exclusion criterion. Of the remaining 184 patients, 56 were barred from analysis for a less-than-14-day ICU stay (because of later admission or a transfer out of ICUs within 14 days, which fails the requirements of continuous observation). Later we sifted out 24 more owing to the limited concentration range of R&D sTREM-1 assay kit. Finally, this study had 104 subjects involved, which included 16 cases of SIRS and 88 cases of sepsis. According to the study guidelines, sepsis patients were further divided into a sepsis group (35 cases) and a severe sepsis group (25 with severe sepsis and 28, septic shock). Of the 88 people, 19 developed AKI upon or 24 h after ICU admission, and 17 developed it more than 48 h after admission, while the remaining 52 were safe from AKI during the observation.

Table 1 provides a general summary of the subjects' conditions. WBC counts and serum PCT levels in the severe sepsis group were higher than that of the sepsis group (*P *= 0.002, *P *< 0.001). There were statistically significant differences in urine sTREM-1 level and disease severity scores, including APACHE II and SOFA scores, among the three groups of patients upon ICU admission (*P *< = 0.001). In terms of kidney function, the SCr and BUN levels for the severe sepsis group tended to be higher than sepsis group (*P *< 0.01). The number of severe sepsis patients that required CRRT was greater than for the sepsis group (*P *= 0.017). More patients developed AKI in the severe sepsis group (*P *< 0.001). Regarding the 28-day survivals, mortality rates for the severe sepsis group were the highest, followed by the sepsis group, and the SIRS group had the lowest rate (*P *< 0.001). Statistically, no significant cross-group difference exists in terms of age, gender, serum CRP, urine output, mechanical ventilation, etiological factors, or accompanying underlying diseases.

**Table 1:** Clinical and biological data at admission according to the guideline for diagnosis of sepsis

Characteristics	SIRS	Sepsis		
	N = 16	total n = 88	sepsis n = 35	severe sepsis n = 53
Age (years)	51 ± 18	60 ± 19	58 ± 21	62 ± 17
Gender (n, %)				
Male	3 (19)	23 (26)	24 (69)	41 (77)
Female	13 (81)	65 (74)	11 (31)	12 (24)
WBC counts (×10^∧^9/L)	11.3 ± 2.8	12.9 ± 6.7	10.5 ± 3.9	14.4 ± 7.7^#^
Serum CRP (mg/dl)	11.4 (5.9, 15.2)	9.5 (4.1, 17.2)	8.1 (2.5, 14.7)	11.8 (6.1, 18.6)
Serum PCT (ng/ml)	1.2 (0.3, 9.5)	2.7 (0.4, 11.1)	0.4 (0.1 to 2.1)	4.5 (1.2 to 19.4)^#^
Urine sTREM-1 (pg/ml)	11.8 (3.6, 24.1)	61.5 (21.3, 173.7)*	28.0 (10.5, 78.8)	83.3 (39.6, 259.7)^#^
Renal characteristics				
Urinary output (ml/Kg/hr)	-	1.2 ± 0.6	1.3 ± 0.5	1.1 ± 0.7
SCr (μmmol/L)	83.2 (69.25, 104.75)	85 (60.5, 134.4)	65.9 (48.0, 99.9)	90.8 (64.4, 167.7)^#^
BUN (mmol/L)	7.9 (4.9, 13.2)	10.4 (7.5, 16.9)	8.0 (6.5, 10.6)	12.7 (8.8 (28.9))^#^
APACHE II score	11.5 ± 7.6	17.5 ± 7.5*	13.4 ± 6.1	20.2 ± 7.1^#^
SOFA score	-	8.6 ± 3.7	5.8 ± 2.9	10.0 ± 3.5^#^
MV (n, %)	12 (75.0)	74 (86.0)	29 (82.9)	45 (84.9)
CRRT (n, %)	2 (12.5)	25 (28.4)	5 (14.3)	20 (37.7)^#^
AKI (n, %)	2 (12.5)	36 (39.8)*	4 (11.4)	31 (58.5)^#^
Etiological factors (n, %)				
Pulmonary infection	-	75 (85.2)	31 (88.6)	44 (83.0)
Abdominal infection	-	22 (25.0)	7 (20.0)	15 (28.3)
Urinary tract infection	-	25 (28.4)	13 (37.1)	12 (22.6)
Trauma/postoperative infection	-	33 (37.5)	10 (28.6)	23 (43.4)
Bacteremia	-	19 (21.6)	9 (25.7)	10 (18.9)
Catheter-related infections	-	13 (14.8)	8 (22.9)	5 (9.4)
Others	-	4 (4.5)	0 (0.0)	4 (7.5)
Underlying diseases (n, %)				
Hypertension	5 (31.2)	32(332 (36.4)	12 (34.3)	20 (37.7)
Diabetes	1 (6.2)	14 (15.9)	5 (14.3)	5 (9.4)
COPD	1 (6.2)	9 (10.2)	5 (14.3)	4 (7.5)
Coronary heart disease	4 (25.0)	17 (19.3)	5 (14.3)	12 (22.6)
Immunosuppressed	0 (0.0)	9(10.2)	2 (5.7)	7 (13.2)
Nervous system disease	2 (12.5)	12(13.6)	5 (14.3)	7 (13.2)
CKD	1 (6.2)	5(5.7)	1 (2.9)	4 (7.5)
Mortality rate (n, %)	1 (6.2)	41(46.6)*	7 (20.0)	34 (64.2)^#^

Quantitative data of normal distribution are presented as mean ± SD. Quantitative data of non-normal distribution are presented as median (25th and 75th percentiles). Qualitative data are presented as n (%). * represents the comparison between SIRS and sepsis, *P *< 0.05; # represents the comparison between sepsis and severe sepsis, *P *< 0.05.

AKI, acute kidney injury; APACHE II score, acute physiologic assessment and chronic health evaluation II scores; BUN, blood urea nitrogen; CKD, chronic kidney disease; CRP, C-reactive protein; CRRT, continuous renal replacement treatment; MV, mechanical ventilation; NS, not significant; PCT, procalcitonin; SCr, serum creatinine; SOFA score, sequential organ failure assessment scores; sTREM-1, soluble triggering receptor expressed on myeloid cells-1; WBC, white blood cells.

### Comparisons of urine sTREM-1, WBC counts, serum CRP, and serum PCT levels, and APACHEII scores: for the purpose of early sepsis diagnosis

**Figure 1:**
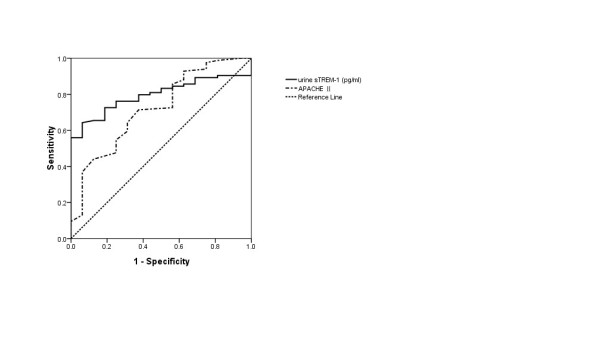
**ROC curves for urine sTREM-1 and APACHEII for distinguishing sepsis from SIRS**. Area under the curve (AUC) turned out respectively as urine sTREM-1 0.797 (95% CI 0.711 to 0.884) and APACHE II score 0.722 (95% CI 0.586 to 0.858). Using a cut-off point for urine sTREM-1 of 34.2 pg/ml, the sensitivity was 0.643, the specificity was 0.938, Positive Predictive Value (PPV) was 0.983, Negative Predictive Value (NPV) was 0.198 and Youden index (YI) was 0.581. Using a cut-off point for APACHE II score of 13.5, the sensitivity was 0.714, the specificity was 0.625, PPV was 0.913, NPV was 0.197 and YI was 0.339. ROC curves, Receiver Operating Characteristic curves;sTREM-1, soluble triggering receptor expressed on myeloid cells-1; APACHEII, acute physiologic assessment and chronic health evaluationII;SIRS, systemic inflammatory response syndrome.

On the first day of enrollment, urine sTREM-1, WBC counts, serum PCT and APACHE II scores of the sepsis group had higher values than the SIRS group (Table 1). However, only differences in urine sTREM-1 level and APACHE II score between the groups proved statistically significant. Figure 1 shows the Receiver Operating Characteristic curves (ROCs) of urine sTREM-1 and APACHE II for sepsis diagnosis.

### Dynamic changes in urine sTREM-1, WBC counts, serum CRP and serum PCT levels: for the assessments of the severity and prognosis of sepsis

**Figure 2:**
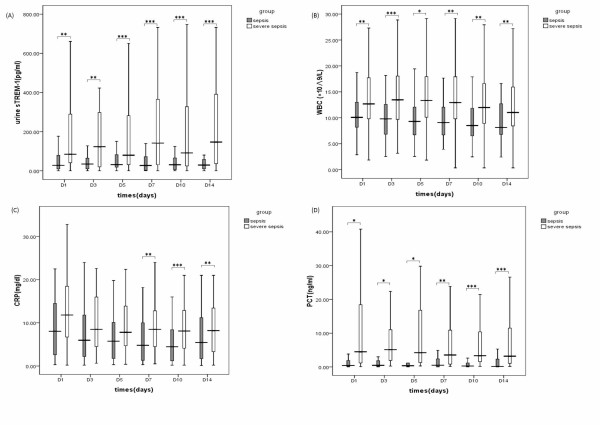
**sTREM-1 (A), WBC (B), CRP (C), and PCT (D) levels measured over 14 days at different stages of sepsis**..*P < 0.05, **P < 0.01, ***P < 0.001. CRP, C-reactive protein; PCT, procalcitonin; sTREM-1, soluble triggering receptor expressed on myeloid cells-1; WBC, White blood cells.

Figure 2 shows comparisons between the sepsis and severe sepsis groups for urine sTREM-1, WBC counts, serum CRP and serum PCT on days 1, 3, 5, 7, 10, and 14. The differences in urine sTREM-1, WBC counts and serum PCT levels at these six different time points were statistically significant, with the severe sepsis group having all-time higher values, and also showing a higher CRP level on days 5, 7, and 10, which were also statistically significant.

**Figure 3:**
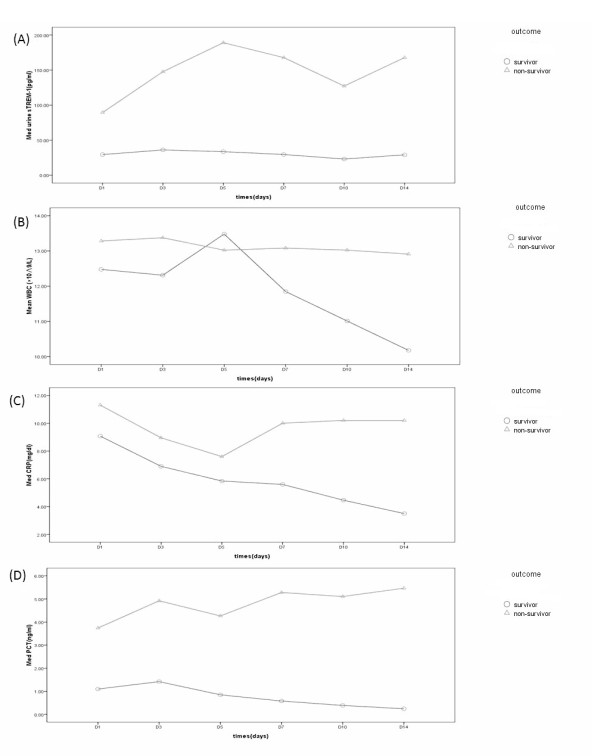
**sTREM-1 (A), WBC (B), CRP (C), and PCT (D) levels measured over 14 days based on 28-day survival**. The differences of patients diagnosed with sepsis in urine sTREM-1 and serum PCT levels at these six different time points were statistically significant (*P *< 0.05), with the non-survivors group having higher values at all time points, and also showing a higher CRP level on days 7, 10 and 14, which were also statistically significant (*P *< 0.05). WBC counts in the non-survivors group were also higher than that of the survivors group, but with no apparent difference between them at all time points. CRP, C-reactive protein; PCT, procalcitonin; sTREM-1, soluble triggering receptor expressed on myeloid cells-1; WBC, white blood cells.

Based on 28-day survivals, the 88 sepsis patients were divided into a survivor group (n = 47) and a non-survivor group (n = 41). Figure 3 compares these two groups for their dynamic changes in urine sTREM-1, WBC counts, serum CRP, and serum PCT levels. The number of survivors decreased with time (41, 41, 32, 25, 13 and 7 people on days 1, 3, 5, 7, 10, and 14 respectively). Therefore, for those who died within 14 days, indicator values obtained at the last test before death were substituted for indicator values at later times after death. The curves in Figure 3 show that the non-survivor group had higher urine sTREM-1, WBC counts, serum CRP and serum PCT levels than the survivor group over this period of time. As for non-survivors, their urine sTREM-1 and serum PCT levels increased with the passage of time, while their WBC counts and serum CRP levels tended to decline. The survivor group exhibited no obvious changes in urine sTREM-1 levels, while their WBC counts, serum CRP, and serum PCT levels were obviously on the decline.

### Urine sTREM-1: diagnostic value for AKI

Of the 88 severe sepsis cases, 19 developed AKI upon or within 24 h of their ICU admission, and 17 developed AKI 48 h later. The other 52 did not develop AKI complications during the time of observation. Table 2 shows the results for the 17 AKI patients who developed AKI before 48 h diagnosis of AKI, such as urine sTREM-1, urine output, CCr, SCr and BUN. Table 2 also shows these indicators' levels upon ICU admission for the 52 patients that did not develop complications. Comparisons were made between these two groups of patients. The AKI patients' urine sTREM-1 levels at 48 h before diagnosis were significantly higher than those for the non- AKI patients (*P *< 0.001). The AKI patients' CCr, SCr and BUN levels at 48 h before diagnosis were also higher than for the non-AKI patients (*P *< 0.05).

**Table 2:** Comparison diagnostic value of sTREM-1, urinary output, CCr, SCr, BUN levels for AKI before 48 hours

Parameters	AKI	Non- AKI	*P*-value
	N = 17	N = 52	
urine sTREM-1 (median-25th and 75th percentiles, pg/ml)	288.74 (108.65, 524.07)	35.66 (7.92, 75.73)	< 0.001
urinary output (mean ± SD, ml/Kg/hr)	1.28 ± 0.62	1.62 ± 0.70	0.086
CCr (mean ± SD, ml/minute)	55.59 ± 30.95	104.28 ± 60.94	0.002
Scr (median-25th and 75th percentiles, μmmol/L)	105.0 (71.7, 176.15)	65.0 (52.18, 90.98)	0.001
BUN (median-25th and 75th percentiles, mmol/L)	17.53 (8.02, 29.73)	8.97 (6.66, 12.43)	0.038

sTREM-1, soluble triggering receptor expressed on myeloid cells-1;CCr, creatinine clearance; SCr, serum creatinine; BUN, blood urea nitrogen; AKI, acute kidney injury.

Multivariate logistic regression was used to assess possible risk factors for AKI occurrence. The variables taken into consideration here include severity of sepsis, urine output, CCr, SCR, BUN, urine sTREM-1 and a history of chronic kidney disease. First, from univariate analyses, four variables were selected for multivariate regression: severity, CCr, SCR and urine sTREM-1. Finally, only urine sTREM-1 and severity were entered into the regression equation. Urine sTREM-1 with a regression coefficient = 0.02, OR = 1.02 and Wald coefficient = 5.246 (*P *= 0.022). Severity with a regression coefficient = 2.074, OR = 7.956 and Wald coefficient = 5.204 (*P *= 0.023).

**Figure 4:**
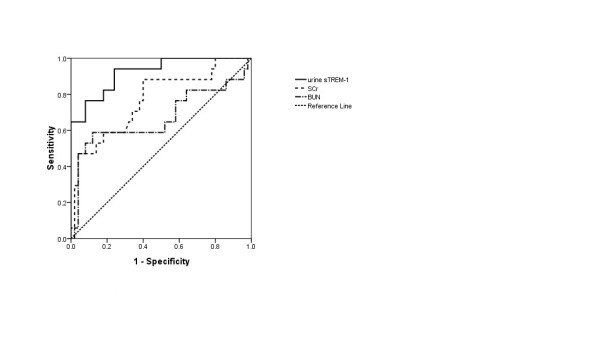
**ROC curves for urine sTREM-1, SCr, BUN level for diagnosing AKI before 48 hours**. ROC curves, Receiver Operating Characteristic curves;sTREM-1, soluble triggering receptor expressed on myeloid cells-1; SCR, serum creatinine; BUN, blood urea nitrogen; AKI, acute kidney injury.

Figure 4 shows ROC curves for AKI diagnosis for urine sTREM-1, SCR, and BUN levels. The corresponding AUCs were as Table 3. Using a cut-off point of 69.04 pg/ml for sTREM-1, the sensitivity turns out 0.941 and the specificity, 0.760.

**Table 3:** Area under ROC curves as means of diagnosing AKI before 48 hours

Variable	AUC	Std. Error	*P*-value	Asymptotic 95% Confidence Interval		Cut	sen	S pe	PPV	NPV	YI
				Lower limit	Upper limit	point					
**Urine sTREM-1**	0.922	0.037	< 0.001	0.85	0.994	69.04	0.941	0.76	0.656	0.964	0.701
**CCr**	0.212	0.061	< 0.001	0.093	0.331	-	-	-	-	-	-
**SCr**	0.776	0.068	0.001	0.644	0.909	68.3	0.882	0.6	0.517	0.911	0.482
**BUN**	0.685	0.091	0.024	0.507	0.864	15.23	0.588	0.88	0.704	0.815	0.468

ROC curves, receiver operating characteristic curves; AKI, acute kidney injury;BUN, blood urea nitrogen; CCr, creatinine clearance; SCr, serum creatinine; sTREM-1, soluble triggering receptor expressed on myeloid cells-1.

## Discussion

Sepsis is a quite common cause of critical diseases and complications, and one of the risk factors for AKI and MODS [28]. When an infection occurs, lipopolysaccharides, lipopeptides, microbial DNA, peptide polysaccharides, lipoteichoic acid and other substances can trigger innate immune reactions mediated by Toll-like receptors (TLRs), NOD-like receptors (NLRs), and other relevant molecules, such as MyD88, and so on. These can subsequently engender both humoral and cellular immune responses. Through the actions of various signals, numerous cytokines, metabolic products of arachidonic acid, leukotrienes, thrombosis-forming substances and inflammatory mediators are produced. In addition, complement and coagulation pathways are activated [29,30]. In case of sepsis, systemic inflammatory factor-induced immune reactions, such as innate and adaptive immune responses, play a critical role in the induction and development of organ dysfunctions, such as AKI [23,31]. This may result from increased expressions of TLRs and NLRs on renal tubule epithelia as well as local endothelial cells inside the kidney. This can cause localized inflammatory reactions in the kidney and oxidative stress, as well as inducing epithelial and endothelial cell injury, and induce changes in the glomerular pore diameter and charge barrier [24,32]

Our study shows that urine sTREM-1 has a positive predictive value for the early diagnosis of sepsis. We do find that there are statistically significant differences between SIRS and sepsis patients upon their ICU admission in urine sTREM-1 level and APACHE II score. ROC curves analysis displays that the urine sTREM-1 AUC is conspicuously higher than any other indicator and its sensitivity reaches 0.643.

While conducting dynamic assessments of the severity and prognosis of sepsis, we found that a severe sepsis group exhibited higher urine sTREM-1, WBC, and serum PCT level than a sepsis group on days 1, 3, 5, 7, 10, and 14, reckoned from ICU admission (*P *< 0.05). All this points to the value of urine sTREM-1, WBC, and serum PCT, as biomarkers, in the discrimination of severe (severe sepsis + septic shock) accompanied by organ dysfunction. Moreover, the differences in these indices between the two groups were observed even on the first day of ICU admission, which is conducive to an early diagnosis of severe sepsis and a timely intervention.

During the 14 days of observation, the non-survivors' urine sTREM-1 levels continued to climb with the passage of time, while the survivors' remained relatively lower. This suggests that urine sTREM-1 may have connections with the prognosis of the disease, and that its continuous, relatively lower expression indicates that the inflammatory response has been brought under control and prognosis might be better. In contrast, in the case of non-survivors, the sTREM-1 level, possibly owing to positive feedback provided by downstream inflammatory factors, rises continually [33,34]. Because of extracellular partial exfoliation brought about by metalloproteinase proteolysis, TREM-1 gives rise to its 27 kDa soluble protein sTREM-1 [15,35], which suggests the release of additional pro-inflammatory cytokines and mediators as well as a continuous or progressive overactive inflammatory response and a poor prognosis. The non-survivors' WBC counts, serum CRP, and serum PCT levels measured higher than those of the survivors during the corresponding period, suggesting a poor prognosis. However, the non-survivors' WBC count and serum CRP level tended to go down towards the end of the course of the disease. All this suggests that the dynamic changes in urine sTREM-1 and serum PCT may well reflect the body's inflammatory responses and the prognosis of sepsis. These two indicators provide more ideal medical tips than WBC counts and serum CRP, as progressive rise of urine sTREM-1 and serum PCT levels may signify a bad prognosis

We discovered that 48 h before AKI occurrence, in contrast with a non-AKI group, that AKI patients had an obvious increase in urine sTREM-1 (*P *< 0.001). The AUC for an ROC curve was 0.922 (95% CI: 0.850 to 0.995), sensitivity was 0.941% (based on a cut-off point of 69.04 pg/ml), and specificity, 0.76. Although the AKI group exhibited higher SCr and BUN level than the non-AKI group before AKI occurrence, a sharp rise was observed only after AKI occurrence. It, therefore, can be deduced that urine sTREM-1 has its value for early sepsis diagnosis, while the indices of SCR and BUN are more significant for assessing the progress of AKI. For our multivariate logistic regression, urine sTREM-1 and sepsis severity were factors entered into the final regression equation, which suggests that throughout the study period, urine sTREM-1 and severity are to be regarded as possible risk factors and urine sTREM-1 possesses potential significance for the assessment of kidney function.

The present study also has some limitations. (1) The sample size was not sufficiently large, including only 17 cases with AKI diagnosed 48 h after ICU admission. For this reason, no statistical significance was found with regards to whether urine sTREM-1 has relevance to AKIN classification (no data reported in this regard) [27]. (2) Blood and urine samples were collected every other day, and not on a day-to-day basis. Therefore, no assessments could be made of kidney function 24 h before AKI occurrence. It cannot be ruled out that renal function decline already existed before AKI diagnosis. (3) There are reports that there are no obvious changes in urine sTREM-1 levels with urinary tract infections [36]. It is, therefore, predicted that a rise in urine sTREM-1 is an inflammatory factor-induced localized kidney immune response brought on by sepsis, not solely by localized inflammatory responses [23,31]. On account of this, the present study does not involve cases with urinary tract infection. (4) In our study, the average CRRT duration for AKI patients measures 11.6 ± 3.8 h per day. In virtue of not receiving continuously renal replacement therapy, the AKI patients in our study might still be featured with a larger quantity of inflammatory factors in their body. In consideration of the sample size, we did not exclude those who received CRRT from this study.

## Conclusions

Urine sTREM-1 may play a role in the early diagnosis of sepsis. The dynamic change in urine sTREM-1 may be an aid to distinguish severity of sepsis, and be more accurate and sensitive than traditional indicators for the dynamic assessments of prognosis. We find that urine sTREM-1 has significance in the early diagnosis of sepsis-related AKI, and could likely become a new marker for such injuries. Owing to the limitations of our sample size, prospective clinical studies are still wanted to provide further proof for the clinical diagnostic value of urine sTREM-1. In addition, further studies are to be expected on the role and mechanisms of urine sTREM-1 for AKI.

## Key messages

• Identification of infection on classic biomarkers is insufficient. But urine sTREM-1 may turn out the most reliable marker for sepsis and its severity.

• Dynamic changes in urine sTREM-1 are more accurate and sensitive than traditional indicators for the dynamic assessments of severity and prognosis of sepsis.

• Urine sTREM-1 can also provide an early warning of possible secondary AKI in sepsis patients. It is likely to be a risk factor and has potential significance for the assessment of kidney function.

• Urine sTREM-1 is likely to become a biomarker with optimistic prospects of clinical application.

## Abbreviations

AKI: acute kidney injury; AKIN: Acute Kidney Injury Network; APACHE II score: Acute Physiologic Assessment and Chronic Health Evaluation II scores; AUC: areas under curve; BUN: blood urea nitrogen; CCr: creatinine clearance; CI: confidence interval; CKD: chronic kidney disease; CRP: C-reactive protein; CRRT: continuous renal replacement treatment; MODS: multiple organ dysfunction syndrome; MV: mechanical ventilation; NLRs: NOD-like receptors; PCT: procalcitonin; ROC curves: Receiver Operating Characteristic curves; SCr: serum creatinine; SIRS: systemic inflammatory response syndrome; SOFA score: Sequential Organ Failure Assessment scores; sTREM-1: soluble triggering receptor expressed on myeloid cells-1; TLRs: Toll-like receptors; WBC: white blood cells.

## Competing interests

The authors declare that they have no competing interests.

## Authors' contributions

LS designed the study, carried it out in the SICU, performed the data analysis, and wrote the first manuscript draft. LF and PY carried out the study in the EICU and the RICU, respectively. JZ and YJ conceived the initial idea for using sTREM-1 levels for infectious diseases and supplemented the study design. DF guided the data analysis and the use of medical statistics. LX was responsible for protocol revisions, data analysis, and final draft revision. All authors have read and approved the final manuscript for publication.
